# Sequencing and analysis of the complete mitochondrial genome in *Anopheles sinensis* (Diptera: Culicidae)

**DOI:** 10.1186/s40249-017-0362-7

**Published:** 2017-10-02

**Authors:** Kai Chen, Yan Wang, Xiang-Yu Li, Heng Peng, Ya-Jun Ma

**Affiliations:** 1 Department of Tropical Infectious Diseases, Second Military Medical University, Shanghai, 200433 China; 2 Department of Medical Microbiology and Parasitology, Second Military Medical University, Shanghai, 200433 China; 3 Team ten Cadet Brigade, Second Military Medical University, Shanghai, 200433 China

**Keywords:** *Anopheles sinensis*, Mitochondrial genome, Phylogenetic relationship

## Abstract

**Background:**

*Anopheles sinensis* (Diptera: Culicidae) is a primary vector of *Plasmodium vivax* and *Brugia malayi* in most regions of China. In addition, its phylogenetic relationship with the cryptic species of the Hyrcanus Group is complex and remains unresolved. Mitochondrial genome sequences are widely used as molecular markers for phylogenetic studies of mosquito species complexes, of which mitochondrial genome data of *An. sinensis* is not available.

**Methods:**

*An. sinensis* samples was collected from Shandong, China, and identified by molecular marker. Genomic DNA was extracted, followed by the Illumina sequencing. Two complete mitochondrial genomes were assembled and annotated using the mitochondrial genome of *An. gambiae* as reference. The mitochondrial genomes sequences of the 28 known *Anopheles* species were aligned and reconstructed phylogenetic tree by Maximum Likelihood (ML) method.

**Findings:**

The length of complete mitochondrial genomes of *An. sinensis* was 15,076 bp and 15,138 bp, consisting of 13 protein-coding genes, 22 transfer RNA (tRNA) genes, 2 ribosomal RNA (rRNA) genes, and an AT-rich control region. As in other insects, most mitochondrial genes are encoded on the J strand, except for ND5, ND4, ND4L, ND1, two rRNA and eight tRNA genes, which are encoded on the N strand. The bootstrap value was set as 1000 in ML analyses. The topologies restored phylogenetic affinity within subfamily Anophelinae. The ML tree showed four major clades, corresponding to the subgenera *Cellia*, *Anopheles*, *Nyssorhynchus* and *Kerteszia* of the genus *Anopheles*.

**Conclusions:**

The complete mitochondrial genomes of *An. sinensis* were obtained. The number, order and transcription direction of *An. sinensis* mitochondrial genes were the same as in other species of family Culicidae.

**Electronic supplementary material:**

The online version of this article (10.1186/s40249-017-0362-7) contains supplementary material, which is available to authorized users.

## Multilingual abstracts

Please see Additional file 1 for translations of the abstract into the six official working languages of the United States.

## Background

*Anopheles sinensis* Wiedemann, 1828 is an oriental species with a wide distribution in China [1]. It is a vector of *Plasmodium vivax* in plain regions of central China, and certain worms such as *Brugia malayi* that cause lymphatic filariasis [2, 3]. Despite its disputable malaria vector capacity, *An. sinensis* is still incriminated as a competent vector for *Plasmodium vivax* malaria due to its abundant population size and wide distribution, which have led to occasional local malaria epidemics or outbreaks throughout history [4].

*Anopheles sinensis* is one of the members in the Hyrcanus Group. The Hyrcanus Group is an extremely complex species assemblage of the genus *Anopheles* subgenus *Anopheles*, which includes above 20 closely related species in China [1, 5, 6]. Because of similar morphological characters of female adult, the identification of these species in the group has been taxonomically problematic. Such as, *An. sinensis* was almost impossible distinguished from its sibling species (*An. lesteri, An. yatsushiroensis, An. kleini,* and so on) [1]. The PCR assay was established by sequences of second internal transcribed spacer (ITS2) region of the ribosomal DNA (rDNA) to identify *An. sinensis* from its cryptic species members of Hyrcanus Group [5]. The genetic structure of *An. sinensis* populations in China were also detected by molecular markers, and the weak genetic structure may be a consequence of low genetic differentiation and high gene flow among populations in central China [7–10]. However, there are still some issues to be elucidated in the molecular classification of *An. sinensis*, such as natural hybrid between *An. kleini* and *An. sinensis* was discovered in the Republic of Korea [11, 12] and China (unpublished data). So, the evolutionary relationship of *An. sinensis*, such as speciation and other issues need to be elucidated further.

Mitochondrial genomes strictly follow maternal inheritance in structure and evolution, and contain abundant information for population genetic and phylogenetic studies [13–21]. There is no adequate mitogenome information available for *An. sinensis*. In this study, we used next-generation sequencing to characterize the mitochondrial genomes of *An. sinensis*, and to reconstruct phylogenetic tree of the known *Anopheles* species.

## Methods

### Mosquito collection and species identification

Wild mosquito adults were collected by CDC mini light traps (BioQuip, USA) or artificial catching aspirator at livestock corrals from Jining and Caoxian County in Shandong Province, China in July, 2012. With the owners’ consent, the light traps were set up in cow pens from 18:30 pm to 8:30 am next day. Mosquitoes of the *An. hyrcanus* group were sorted out in the field by morphology using the identification keys [1], and kept individually in silica gel filled tubes at 4 °C until DNA extraction. After being brought back to the laboratory, the female adults were separated into head and body. The single head was used to identify species by PCR assay based on rDNA ITS2 sequences [5]. Twenty bodies pool of *An. sinensis* species was extracted genomic DNA using Meta-G-Nome™ DNA Isolation Kit (Epicentre, USA), followed by Illumina sequencing.

### Metagenome sequencing

Genomic DNA was fragmented and sequencing libraries were prepared, which insert size was about 700 bp. Double-end pairing sequencing was performed using Illumina HiSeq 2000 (Genewiz, USA). Each library was sequenced to generate about 20 million paired-end reads from each sample. The mean length of reads was 101 base pairs.

### Mitochondrial genome assembly and annotation

The quality of total reads was controlled by FASTQC (http://www.bioinformatics.babraham.ac.uk/projects/fastqc/), then the high quality reads were mapping to *An. sinensis* genome (SAMN02910229) [22]. The contigs were assembled by KmerGenie softwares [23], and aligned with mitochondrial genome of Culicidae mosquitoes on GenBank BLAST website, which identity threshold value was set as 90%. The complete mitochondrial genome of *An. sinenesis* was obtained, including 13 protein coding genes, 22 transfer RNA (tRNA) genes, 2 ribosomal RNA (rRNA) genes and AT-rich control region. The genes and region of the mitochondrial genome were identified by comparison with the reference mitogenome sequences of *An. gambiae* (GenBank Accession No. L20934.1) and other *Anopheles* mosquitoes. Some parts fragments such as CO1 and CO2 region were verified by PCR products [7, 24].

### Phylogenetic analysis

A total of 28 *Anopheles* species (including *An. sinensis* in this study) mitochondrial genome sequences from NCBI database available were analyzed by MEGA 6 software [25]. The phylogenetic relationships of the mitogenome DNA sequences for *Anopheles* mosquitoes were reconstructed using Maximum Likelihood (ML) method with PhyML 3.0 [26]. The best fit model of nucleotide substitution, the *GTR + I + G* model, was determined for the ML tree inference with Modeltest 3.7 [27]. The bootstrap values for 1000 replicates were calculated.

## Results and discussion

### Mitogenome organization and composition

The length of two complete mitochondrial genomes of *An. sinensis* were obtained from two samples, which were 15,076 bp and 15,138 bp, respectively. Both sequences was conserved, except the length of AT-rich control region. One of the complete mitochondrial genome sequence was chosen for further analysis, which was submitted to GenBank (Accession No. KT218684.1).

**Fig. 1:**
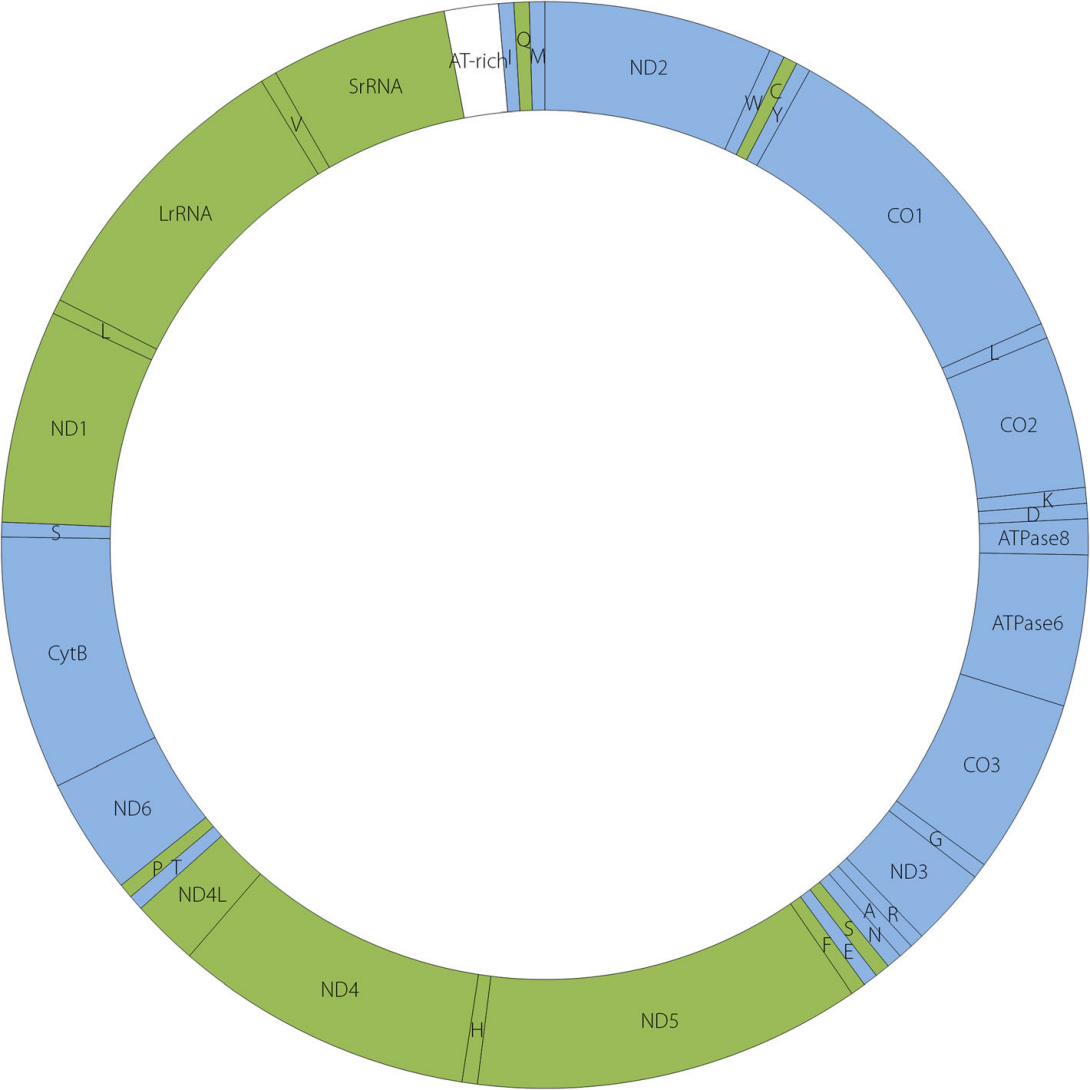
Graphic representation of the gene arrangement and gene order of the mitochondrial genome of *An. sinensis*. All 13 protein-coding genes, 22 tRNA genes, 2 rRNA genes, and the AT-rich control region are indicated in the circle. Each tRNA gene is identified by its single letter abbreviation. The direction of transcription is indicated by colour, the genes in blue content (J strand) as clockwise and in green (N strand) as counterclockwise

The mitochondrial genomes of *An. sinensis* mosquitoes consisted of circular DNA molecules, contained 13 protein-coding genes, 22 transfer RNA (tRNA) genes, 2 rRNA genes (12S rRNA and 16S rRNA), and an AT-rich control region (Fig. 1). The mitochondrial genes showed no length variation on either the J or N strand. The AT-rich control region was located between the SrRNA and tRNA-Ile genes.

### Protein coding genes

The mitochondrial genome of *An. sinensis* species consisted of 13 intron-less protein coding genes. The gene number, order and transcription direction of mitochondrial genes were the same as in other species of Culicidae species [13–15, 17, 19, 21, 28–33]. There were 9 genes located in the J strand, as ND2, CO1, CO2, ATPase8, ATPase6, CO3, ND3, ND6 and CytB, while ND1, ND5, ND4 and ND4L located in the N strand. The length of nucleotides and amino acids of these 13 genes were showed in Table 1.

**Table 1:** Protein-coding genes information of mitochondrial genome of *An. sinensis*

Gene name Code	Length of nucleotides (bp)	Length of amino acids	Encoded polypeptide
ND2	1023	341	NADH dehydrogenase subunit 2
CO1	1539	513	cytochrome c oxidase subunit I
CO2	684	228	cytochrome c oxidase subunit II
ATPase8	159	53	ATP synthase subunit 8
ATPase6	678	226	ATP synthase subunit 6
CO3	786	262	cytochrome c oxidase subunit III
ND3	351	117	NADH dehydrogenase subunit 3
ND5	1737	579	NADH dehydrogenase subunit 5
ND4	1341	447	NADH dehydrogenase subunit 4
ND4L	300	100	NADH dehydrogenase subunit 4 L
ND6	522	174	NADH dehydrogenase subunit 6
CytB	1134	378	cytochrome b
ND1	954	318	NADH dehydrogenase subunit 1

### Transfer and ribosomal RNAs genes

All 22 tRNA genes were dispersed in the mitochondrial genome of *An. sinensis*, the length and position of them was similar with the reported Culicidae mosquitoes (Table 2) [13–15, 20, 21, 28, 29, 31–33]. Among them, there were two kinds of transfer genes for serine and leucine, and the remaining 18 tRNA genes correspond to the other amino acid respectively. The arrangement of tRNA-Arg and tRNA-Ala was inverse compared with the insects of family Drosophilidae, Tephritidae, Calliphoridae, Muscidae and Ceratopogonidae [34–36].

**Table 2:** tRNA genes information of mitochondrial genome of *An. sinensis*

tRNA gene (Code)	Length of nucleotide (bp)	Anticodon	Location (strand)
tRNA-Ile (I)	68	GAU	J
tRNA-Gln (Q)	68	UUG	N
tRNA-Met (M)	69	CAU	J
tRNA-Trp (W)	69	UCA	J
tRNA-Cys (C)	63	AUU	N
tRNA-Tyr (Y)	65	UAA	J
tRNA-Leu (L)	66	UUA	J
tRNA-Lys (K)	71	UAA	J
tRNA-Asp (D)	68	GUC	J
tRNA-Gly (G)	68	UCC	J
tRNA-Arg (R)	64	UCG	J
tRNA-Ala (A)	66	UGC	J
tRNA-Asn (N)	69	GUU	J
tRNA-Ser (S)	67	UUA	N
tRNA-Glu (E)	66	UUC	J
tRNA-Phe (F)	67	GAA	N
tRNA-His (H)	69	GUG	N
tRNA-Thr (T)	68	UGU	J
tRNA-Pro (P)	66	GAG	N
tRNA-Ser (S)	66	UGA	J
tRNA-Leu (L)	66	UAG	N
tRNA-Val (V)	72	AUU	N

There were 2 rRNA genes in mitochondrial genome of *An. sinensis*, both located in the N strand. The length of SrRNA gene was 692 bp encoding 12S rRNA, and the LrRNA gene was 1329 bp encoding 16S rRNA.

### Phylogenetic analysis

**Fig. 2:**
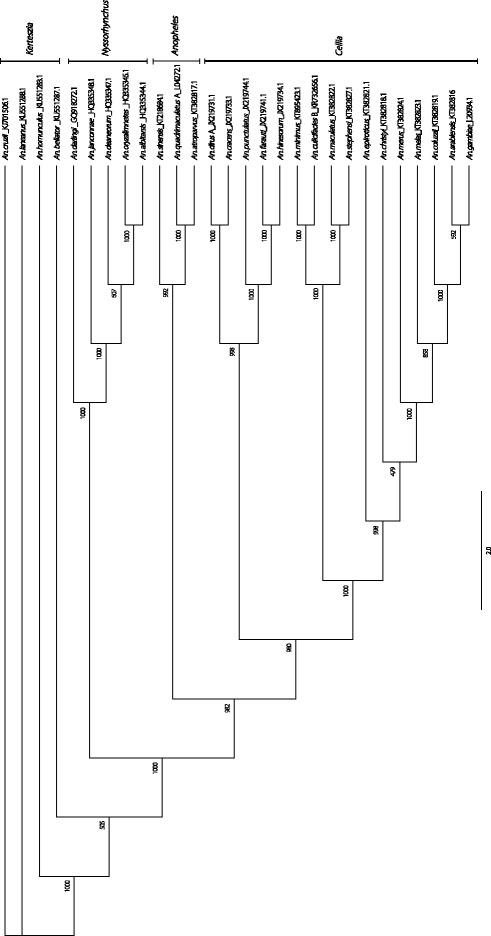
Phylogenetic tree generated using the Maximum Likelihood method based on complete mitochondrial genomes. The bootstrap values are marked on each node of the tree. The GenBank Accession No. is in bracket: *An. sinensis* (KT218684.1), *An. arabiensis* (KT382816), *An. quadrimaculatus* A (L04272.1), *An. darlingi* (GQ918272.1), *An. atroparvus* (KT382817.1), *An. minimus* (KT895423.1), *An. dirus* A (JX219731.1), *An. coluzzii* (KT382819.1), *An. oryzalimnetes* (HQ335345.1), *An. albitarsis* (HQ335344.1), *An. culicifacies* B (KR732656.1), *An. epiroticus* (KT382821.1), *An. gambiae* (L20934.1), *An. farauti* (JX219741.1), *An. deaneorum* (HQ335347.1), *An. merus* (KT382824.1), *An. melas* (KT382823.1), *An. janconnae* (HQ335348.1), *An. hinesorum* (JX219734.1), *An. cracens* (JX219733.1), *An. maculatus* (KT382822.1), *An. stephensi* (KT382827.1), *An. punctulatus* (JX219744.1), *An. christyi* (KT382818.1), *An. cruzii* (KJ701506.1), *An. laneanus* (KU551288.1), *An. homunculus* (KU551283.1), *An. bellator* (KU551287.1)

The molecular phylogenetic relationship using complete mitochondrial genome sequence of 28 *Anopheles* species was analyzed. All sequences generated in this study have been deposited in the GenBank. The topologies restored phylogenetic affinity within subfamily Anophelinae (Fig. 2). The ML tree showed that two major clades with mosquitoes in subgenus *Kerteszia* (*n* = 4) and the other three subgenera, as *Cellia* (*n* = 16)*, Anopheles* (*n* = 3)*, Nyssorhynchus* (*n* = 5) in genus *Anopheles*. The latter subgenera *Cellia, Anopheles* and *Nyssorhynchus* were monophyly, and the subgenera *Cellia* with *Anopheles* was sister relationship. The bootstrap values were almost above 50%.

## Conclusions

The complete mitochondrial genomes of *An. sinensis* were obtained. The number, order and transcription direction of *An. sinensis* mitochondrial genes were the same as in other species of Culicidae. And the complete mitogenome data can provide basic information for analyzing phylogenetic relationship of mosquito species.

## Additional file

Additional file 1: Multilingual abstracts in the six official working languages of the United Nations. (PDF 666 kb)

## Abbreviations

ITS2: Second internal transcribed spacer
ML: Maximum likelihood
rDNA: Ribosomal DNA
rRNA: Ribosomal RNA
tRNA: Transfer RNA
