# Supplementary material for: Sequencing and analysis of the complete mitochondrial genome in Anopheles sinensis (Diptera: Culicidae)
Source: Infect Dis Poverty. 2017 Oct 2;6:149. doi: 10.1186/s40249-017-0362-7 (PMC5625653; doi:10.1186/s40249-017-0362-7)
Supplement: Additional file 1: — Multilingual abstracts in the six official working languages of the United Nations. (PDF 666 kb) [file 40249_2017_362_MOESM1_ESM.pdf]

Translation of the abstract into the six official working languages of the United Nations

تسلسل وتحليل الجينوم المتقدِّريّ الكامل في بعوضة الملاريا الصينية (ذوات الجناحين: البعوضيات)

كاي تشن، ويان وانغ، وشيانغيو لي، وهنغ بنغ، وياجون ما

ملخص
لمحة عامة: بعوضة الملاريا الصينية (ذوات الجناحين: البعوضيات) هي ناقل أساسي لـالمتصورة النشيطة و البروجِيةُ المَلاوية ي معظم أنحاء الصين. وبالإضافة إلى ذلك، تعد علاقة نشوءها وتطورها مع الانواع الخفية من المجموعة الهيركانيّة معقدة ولا تزال دون حل. ويستخدم تسلسل الجينوم المتقدِّريّ على نطاق واسع كعلامات جزيئية لدراسات نشوء وتطور مجمعات أنواع البعوض، ويعد منها بيانات الجينوم المتقدِّريّ لـبعوضة الملاريا الصينية غيرالمتاحة.
الأساليب: بعوضة الملاريا الصينية تم جمع العينات من شاندونغ، في الصين، والتي تم تحديدها من قبل علامة الجزئية. تم استخراج الحمض النووي الجيني، يليه تسلسل إلومينا. تم تجميع اثنين من الجينومات المتقدِّريّة كاملة و شرحت باستخدام الجينوم المتقدِّريّ لـ بعوضة الملاريا الغامبية كمرجع. تم محاذاة تسلسل الجينوم المتقدِّريّ لـ 28 بعوضة ملاريامعروفة الأنواع وأعيد بناء الشجرة التطورية بواسطة أسلوب الاحتمال الأقصى (ML).
النتائج:طول الجينومات المتقدِّريّة كاملةً لـ بعوضة الملاريا الصينية كان 15 076 نقطة أساس و 15 138 نقطة أساس، ويتألف من 13 جين ترميز للبروتين، و 22 جين نقل الحمض النووي الريبي (الحمض الريبي النووي النقال)، 2 الجينات الريباسي (الرنا الريباسي)، و والمنطقة التي يزيد فيها سيطرة الأدينين و الثيمين. . كما هو الحال في الحشرات الأخرى، يتم ترميز معظم الجينات المتقدِّريّة على طاق J، باستثناء ND5، ND4، ND4L، ND1، واثنان رنا وثماني جينات حمض ريبي نووي نقال، والذين يتم ترميزيهم على طاق N. تم تعيين قيمة التمهيد بـ 1 000 في تحليل ML. فيما استعادت الطبولوجيا تقارب النشوء والتطور داخل فصيلة الأنوفيلينات. وأظهرت شجرة ML أربع فصائل رئيسية، المقابلة لجُنيسات سيليا، بعوضة الملاريا، مائلة الخطم و كرتسيزيامن جنس بعوضة الملاريا.
الاستنتاجات:تم الحصول على الجينومات المتقدِّريّة كاملةً لـ بعوضة الملاريا الصينية كان عدد ونظام والنسخة المكتوبة للجينات المتقدِّريّة لـ بعوضة الملاريا الصينية هي نفسها كما في الأنواع الأخرى من أسرة البعوضيات.

Translated from English version into Arabic by Eman Shahen, through

TRANSLATORS
WITHOUT BORDERS

中华按蚊线粒体基因组全序列测定和分析（双翅目：蚊科）

陈锴，王琰，李翔宇，彭恒，马雅军

摘要
引言：中华按蚊是中国大部分地区间日疟原虫和马来丝虫的主要传播媒介，它与赫坎按蚊种团的其他近缘种进化关系复杂，尚未完全阐明。线粒体基因组序列已被广泛用作蚊种间系统发育关系研究的分子标志，而中华按蚊的线粒体基因组尚未见报告。
方法：在中国山东现场采集按蚊，分子鉴定为中华按蚊后，提取基因组 DNA，并进行 Illumina 高通量测序。以冈比亚按蚊线粒体基因组作为参考，我们组装与注释了两个完整的中华按蚊线粒体基因组，并与 28 种已知按蚊的线粒体基因组序列进行比对，使用最大似然法（Maximum

Likelihood, ML）构建系统发育树。
结果：两个中华按蚊线粒体基因组的全长分别为 15,076 bp 和 15,138 bp，包括 13 个蛋白编码基因，22 个转运 RNA（tRNA），2 个核糖体 RNA（rRNA）基因和一个富含 AT 的控制区（AT-rich）。与其他昆虫相似的是，中华按蚊主要的线粒体基因编码于 J 链，而 ND4、ND5、ND4L、ND1、2 个 rRNA 基因和 8 个 tRNA 位于 N 链。Bootstrap 检验 1000 次的 ML 树显示拓扑关系与按蚊亚科亲缘关系一致，包含 4 个主要分支，分别对应按蚊属的塞蚊亚属、按蚊亚属、覆蚊亚属和 *Kerteszia* 亚属的种类。
结论：本研究获得了中华按蚊的线粒体全基因组，其基因数量、顺序和转录方向与蚊科其他种类相同。

Translated from English version into Chinese by Kai Chen

Séquençage et analyse du génome mitochondrial complet d'*Anopheles sinensis* (Diptera: Culicidae)

Kai Chen, Yan Wang, Xiangyu Li, Heng Peng, Yajun Ma

Résumé
Contexte: *Anopheles sinensis* (Diptera: Culicidae) est un vecteur primaire de *Plasmodium vivax* et *Brugia malayi* dans la plupart des régions de Chine. Sa relation phylogénique avec les espèces cryptiques du groupe Hyrcanus est complexe et irrésolue à ce jour. Les séquences du génome mitochondrial sont largement utilisées comme marqueurs moléculaires pour les études phylogéniques des complexes d'espèces de moustiques. Il n'existe cependant pas de données sur le génome mitochondrial d'*A. sinensis*.
Méthodes: Des spécimens d'*A. sinensis* ont été collectés dans la province chinoise du Shandong et identifiés par un marqueur moléculaire. L'ADN génomique a été extrait et séquencé sur la plateforme Illumina. Deux génomes mitochondriaux complets ont été assemblés et annotés en prenant comme référence le génome mitochondrial d'*A. gambiae*. Les séquences du génome mitochondrial des 28 espèces connues du genre *Anopheles* ont été alignées et l'arbre phylogénique reconstitué par la méthode de probabilité maximale (Maximum Likelihood, ML).
Résultats: La longueur des génomes mitochondriaux complets d'*A. sinensis* était de 15,076 pb et 15,138 pb, avec 13 gènes codant pour des protéines, 22 gènes pour l'ARN de transfert (ARNt), 2 pour l'ARN ribosomique (ARNr) et une région de contrôle riche en AT. Comme chez les autres insectes, la plupart des gènes mitochondriaux sont encodés sur le brin J, sauf ND5, ND4, ND4L, ND1, deux gènes d'ARNr et huit d'ARNt, qui sont codés sur le brin N. La valeur d'autoamorçage (bootstrap) a été fixée à 1000 dans les analyses ML. Les topologies ont reconstitué les affinités phylogénétiques dans la sous-famille des *Anophelinae*. L'arbre de probabilités maximales fait apparaître quatre grands clades correspondant aux sous-genres *Cellia*, *Anopheles*, *Nyssorhynchus* et *Kerteszia* du genre *Anopheles*.
Conclusions: Les génomes mitochondriaux complets d'*A. sinensis* ont été obtenus. Le nombre, l'ordre et le sens de transcription des gènes mitochondriaux d'*A. sinensis* étaient les mêmes que dans les autres espèces de la famille des Culicidae.

Translated from English version into French by Suzanne Assenat, through

Секвенирование и анализ полного митохондриального генома в *Anopheles sinensis* (Двукрылые: Комары)

Кай Чень, Янь Ван, Сиан Ю Ли, Хэн Пэн, Яджунь Ма

Аннотация

Контекст исследования: *Anopheles sinensis* (Двукрылые: Комары) является главным переносчиком *Plasmodium vivax,* а также *Brugia malayi* в большинстве регионов Китая. Кроме того, его филогенетические связи с криптическими видами группы "Anopheles Hyrcanus" сложны и остаются непроясненными. Последовательности митохондриального генома широко используются в качестве молекулярных маркеров для филогенетических исследований комплексов видов комаров, из которых данные митохондриального генома *Anopheles sinesis* недоступны.

Методы: *Образцы* An. sinensis были собраны в Шаньдуне, Китай, и идентифицированы молекулярным маркером. Была извлечена геномная ДНК с последующим секвенированием Illumina. Два полных митохондриальных генома были собраны и отмечены с использованием митохондриального генома *Anopheles gambiae* в качестве образца. Последовательности митохондриальных геномов 28 известных видов *малярийного комара (Anopheles)* были выровнены и реконструированы филогенетическое дерево методом максимального правдоподобия (ML).

Выводы: Длина полных митохондриальных геномов *An. sinensis* составила 15,076 и 15,138 пар нуклеотидных оснований, состоящих из 13 генов, кодирующих белок, 22 гена транспортных РНК (тРНК), 2 гена рибосомных РНК (рРНК) и участки, обогащенные парами аденин-тимин (А-Т). Как и у других насекомых, большинство митохондриальных генов кодируют J-цепь, за исключением ND5, ND4, ND4L, ND1, двух рРНК и восьми генов тРНК, кодирующих H-цепь. При анализе метода максимального правдоподобия значение индекса бутстрепа было установлено в размере 1000. Топологии восстановили филогенетическую близость в подсемействе *Anophelinae*. Дерево ML показало четыре основных клады, соответствующие подроду *Cellia*, *Anopheles*, *Nyssorhynchus,* а также *Kerteszia* рода *Anopheles*.

Заключение: Полные митохондриальные геномы *An. sinensis* были получены. Количество, порядок и направление транскрипции митохондриальных генов *An. sinensis* были такими же, как и у других видов семейства Culicidae.

Translated from English version into Russian by Karina1207, through

Secuenciación y análisis del genoma completo mitocondrial en *Anopheles sinensis* (Diptera: Culicidae)

Kai Chen, Yan Wang, Li Xiangyu, Heng Peng, Yajun Ma

Resumen

Antecedentes: *Anopheles sinensis* (Diptera: Culicidae) es un vector primario de *Plasmodium vivax* y *Brugia malayi* en la mayoría de las regiones de China. Además, su relación filogenética con las crípticas especies del Grupo Hyrcanus es compleja y sigue siendo incierta. Las sequencias de genoma mitocondrial son ampliamente usadas como marcadores moleculares en estudios filogenéticos de complejos de especie del mosquito, de los cuáles no hay datos diponibles del genoma mitocondrial de An. Sinensis.

Metodología: Muestras de *An. Sinensis* de Shandong, China, fueron recogidas y analizadas por marcadores moleculares. Se extrajo ADN genómico, seguido por la secuenciación Illumina. Dos genomas mitocondriales completos fueron ensamblados y anotados tomando el genoma mitocondrial de *An. Gambiae* como referencia. Las secuencias de genomas mitocondriales de 28 especies conocidas de *Anopheles* fueron alineadas y se recreó un árbol filogenético siguiendo el método de Máxima Verosimilitud (MV).

Resultados: La longitud de genomas mitocondriales completos de *An. Sinensis* fue de 15,076 pb y 15,138 pb, integrados por 13 genes codificantes de proteínas, 22 genes de ARN de transferencia (ARNt), 2 genes de ARN ribosómico (ARNr) y una región de control rica en AT. Al igual que en otros insectos, la mayoría de los genes mitocondriales están codificados en la cadena J, excepto ND5, ND4, ND4L, ND1, dos ARNr y ocho genes ARNt, los cuáles están codificados en la cadena N. El valor bootstrap se situó en 1000 en análisis MV. Las topologías restauraron la afinidad filogenética dentro de la subfamilia *Anophelinae.* El árbol MV mostró cuatro clados mayoritarios, correspondientes a los súbgeneros *Cellia*, *Anopheles*, *Nyssorhynchus* y *Kerteszia* del género *Anopheles*.

Conclusiones: Se obtuvieron los genomas mitocondriales completos de *An. Sinensis.* El número, orden y dirección de la transcripción de los genes mitocondriales de *An. sinensis* fueron iguales a los de otras especies de la familia Culicidae.

Translated from English version into Spanish by spinos, through
